# Endocan can be a predictive marker of severity of sepsis but cannot be a marker of acute respiratory distress syndrome in ICU patients

**DOI:** 10.1186/cc14315

**Published:** 2015-03-16

**Authors:** M Mizunuma, H Ishikura, Y Nakamura, K Muranishi, S Morimoto, H Kaneyama, Y Izutani, T Nishida, A Murai

**Affiliations:** 1 Fukuoka University Hospital, Fukuoka, Japan

## Introduction

Endocan (endothelial cell specific molecule-1), a 50 kDa dermatan sulfate proteoglycan, is expressed by endothelial cells in the lung and kidney. It was reported that the serum Endocan level is related to the severity of sepsis and positive correlation with the mortality rate. On the other hand, it was also reported that lower levels of serum Endocan are associated with subsequent development of chronic kidney disease, and chronic liver disease acute lung injury (ALI) in trauma patients. The aim of this study is confirmation of the relationship between serum Endocan level and the severity of sepsis, and also the severity of acute respiratory distress syndrome (ARDS) in septic patients.

## Methods

This study was conducted as a single-center, retrospective, observational study in the emergency department of Fukuoka University Hospital from April 2010 to August 2013. Blood samples were collected within 2 hours when the patients were diagnosed with ARDS. In this time we adopted the Berlin definition as the categorized of ARDS severity. Furthermore, we evaluated the extravascular lung water index (EVLWi) and pulmonary vascular permeability index (PVPI) as a condition of ARDS using the transpulmonary thermodilution method. Additionally, 10 healthy donors were entered as a control. The patients were divided into nonsepsis, severe sepsis and septic shock using the ACCP/SCCM guidelines.

## Results

We enrolled 70 ARDS patients during the investigation periods. We met six patients with nonsepsis, 27 with severe sepsis and 37 with septic shock. The serum Endocan levels were significantly higher in patients with septic shock (3.7 to 3.9 ng/ml) than in patients with severe sepsis (1.7 to 2.3 ng/ml, *P *< 0.05), nonsepsis (0.6 to 0.3 ng/ml, *P *< 0.05) and healthy donors (0.4 to 0.1 ng/ml, *P *< 0.05). However, there was no significant correlation between the Endocan level and the severity of ARDS. In addition, significant correlation between the Endocan level and EVLWi and PVPI was not observed.

## Conclusion

These results suggested that there was good relationship between the Endocan level and the severity of sepsis. But unlike the trauma patients, correlation between the severity of ARDS and Endocan value was not recognized.
